# Biomarkers of Common Molecular Dysregulation in Tumor Tissue and Peritumor Mucosa in Head and Neck SCC: Insights into Field Cancerization

**DOI:** 10.3390/ijms27031212

**Published:** 2026-01-25

**Authors:** Lyuben Dimitrov, Gergana S. Stancheva, Silva G. Kyurkchiyan, Milena Mitkova, Iglika Stancheva, Silviya Valcheva, Kristina Komitova, Silviya Skelina, Julian Rangachev, Todor M. Popov

**Affiliations:** 1 Department of ENT, Medical University, 1527 Sofia, Bulgaria; liuben97@gmail.com (L.D.);; 2 Molecular Medicine Center, Medical University, 1000 Sofia, Bulgariasggiragosyan@abv.bg (S.G.K.);; 3 Department of Neurology, Medical University, 1000 Sofia, Bulgaria

**Keywords:** field cancerization, peritumoral mucosa, molecular margins, biomarkers, head and neck squamous cell carcinoma, laryngeal carcinoma, microRNA, CDKN2A, MDM2, E2F2

## Abstract

Field cancerization is a fundamental paradigm in tumorigenesis, emphasizing that carcinogenesis begins long before the appearance of clinically detectable lesions and often precedes recognizable premalignant changes. A direct manifestation of this process is the molecular dysregulation observed in the peritumoral mucosa—histologically normal-appearing tissue that nonetheless exhibits genetic and epigenetic alterations similar to those of the adjacent tumor. This review summarizes current evidence on the molecular alterations shared between tumor tissue and peritumoral mucosa in HNSCC and evaluates their potential as biomarkers for defining molecular margins and improving surgical precision. A literature search was conducted in PubMed using combinations of the keywords “peritumor,” “laryngeal”, “HNSCC,” and “field cancerization.” Studies were included if they directly compared tumor tissue with peritumoral mucosa and, preferably, a third set of distant normal control samples. Only nine studies met the inclusion criteria, highlighting the scarcity of focused research in this area. Reported biomarkers exhibiting comparable dysregulation in both tumor and peritumor tissues include MDM2, E2F2, CDKN2A/p16, ETS-1, MGMT, and multiple microRNAs (e.g., miR-21, miR-96-5p, miR-145-5p). These molecular signatures demonstrate the presence of a biologically altered field extending beyond histologically defined tumor margins. Peritumoral mucosal dysregulation, as a consequence of field cancerization, underscores the need to redefine surgical margins at the molecular level. The identification and validation of biomarkers reflecting this continuum could enable the establishment of molecular margins—improving risk assessment, reducing local recurrence, and advancing personalized oncologic surgery in HNSCC. Standardizing definitions and sampling protocols for “normal adjacent tissue” remains essential for future translational research.

## 1. Introduction

Field cancerization is a fundamental paradigm in tumorigenesis, emphasizing that carcinogenesis begins long before the appearance of clinically detectable lesions and often precedes recognizable premalignant changes. A direct manifestation of this process is the molecular dysregulation observed in the peritumoral mucosa—histologically normal-appearing tissue that nonetheless exhibits genetic and epigenetic alterations similar to those of the adjacent tumor. This phenomenon highlights that tumorigenesis in head and neck cancers extends beyond the visible tumor mass into a broader, biologically altered field.

Despite decades of recognition, comprehensive and up-to-date syntheses addressing the molecular mechanisms underlying peritumoral dysregulation in head and neck squamous cell carcinoma remain limited. The present review aims to consolidate the existing knowledge and provide an overview of studies that have directly compared molecular alterations in tumor tissue and peritumoral mucosa, ideally including a third set of distant, histologically normal control samples. Such a comparative approach allows for the identification of shared molecular dysregulation between tumor and adjacent non-tumorous tissues and provides critical insights into the continuum of genetic and epigenetic changes underlying field cancerization. By summarizing and critically evaluating all available publications employing this tripartite study design, this review highlights current progress, methodological limitations, and key areas where further research is needed to clarify the mechanisms driving early malignant transformation and local recurrence in head and neck cancer.

The concept of field cancerization was first described in 1953, when pathologic atypia was identified in clinically normal tissues surrounding oropharyngeal carcinomas. Slaughter et al. conducted a landmark study examining histopathology slides from 783 patients with head and neck cancer, revealing that epithelial alterations extended beyond the tumor boundaries in all cases [1]. Notably, approximately 11% of the patients exhibited multiple independent malignant foci. Their findings suggest that the mucosa in the head and neck undergoes widespread molecular and histological changes, likely driven by carcinogen exposure, increasing susceptibility to multifocal malignant transformation. This understanding has shaped modern perspectives on cancer progression, recurrence, and development of secondary primary tumors in head and neck squamous cell carcinoma [1,2,3]. Before the growth of a malignant lesion, a normal cell can acquire protumorigenic genetic mutations that are positively selected in the microenvironment of an otherwise healthy organ.

## 2. Principles and Definition of Field Cancerization

Exposure to carcinogens results in the development of an altered epithelial field, in which multiple independent foci of abnormal cells can emerge, each with the potential to evolve into premalignant or malignant lesions. This concept has long been invoked to explain the occurrence of multiple primary tumors and local recurrences following complete surgical excision.

Field cancerization is traditionally defined as an increased risk of malignant transformation across the entire upper aerodigestive tract, resulting from the accumulation of multiple genetic and epigenetic alterations throughout the mucosal field after prolonged carcinogen exposure [3]. Subsequently, Boudewijn et al. proposed a molecularly oriented definition, describing a field lesion as one or more areas composed of epithelial cells harboring genetic abnormalities. These field lesions are typically of monoclonal origin, lack invasive or metastatic potential, and are considered preneoplastic by nature, often displaying histopathological features of dysplasia [4]. Similarly, Gabriel et al. [5] broadened the definition, describing field cancerization as “the process whereby cells in a particular tissue or organ are transformed such that genetically altered but histologically normal-appearing cells predate the development of neoplasia or coexist with malignant cells, irrespective of clonality”.

In their seminal 1953 study, Slaughter et al. [1] demonstrated that cancer does not arise as an isolated cellular event but rather represents a diffuse process involving many epithelial cells simultaneously. They proposed that repeated carcinogenic insults give rise to widespread epithelial transformation, progressing at variable rates within a single anatomical field. Later, the term lateral cancerization was introduced to describe the local expansion of genetically altered cells adjacent to a tumor, driven by progressive transformation rather than by the direct invasion of existing cancer cells.

At the molecular level, distinguishing between mutant and cancerized cell lineages remains challenging. While somatic mutations accumulate naturally throughout life [6], only a subset of these confer selective advantages—such as increased proliferation, reduced apoptosis, or immune evasion—that promote clonal expansion and malignant potential. Neutral drift among adult stem cells can lead to the dominance of certain mutant clones within aging tissues [7]; however, not all such clones represent true cancerized fields. To address this distinction, Curtius et al. [8] refined the concept, defining a cancerized field as a population of cells that has acquired some, but not all, of the phenotypic hallmarks required for malignancy. These partial phenotypic shifts may occur without overt morphological abnormalities and arise from mutations in key cancer-associated genes, acting either autonomously or in concert with alterations in the surrounding microenvironment. This refined framework provides a more practical and mechanistic understanding of field cancerization, particularly relevant to the study of laryngeal carcinoma, where shared molecular dysregulation between tumor and peritumoral mucosa reflects the biological continuum of carcinogenesis.

## 3. Molecular Findings for the Concept of Field Cancerization

Field cancerization in the head and neck region is driven by a complex cascade of molecular and cellular events. A literature search was performed in PubMed using the keywords “peritumor” OR “laryngeal” AND “field cancerization” OR “HNSCC” AND “field cancerization.” In addition, citation tracking of the seminal article by Slaughter et al. was undertaken. The search encompassed the full range of records indexed in PubMed since its inception. Only peer-reviewed articles published in English were considered.

**Figure 1:**
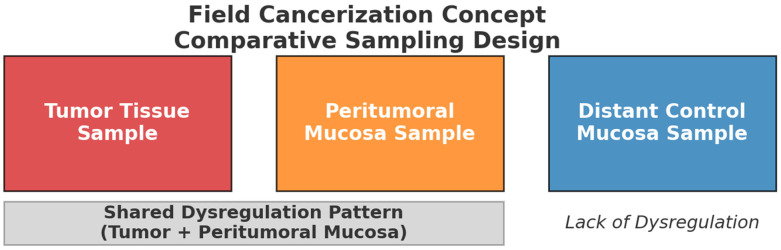
Comparative sampling design for assessing field cancerization. Tumor and peritumoral mucosa samples reveal shared dysregulation patterns, while distant control mucosa serves as a non-dysregulated reference.

**Figure 2:**
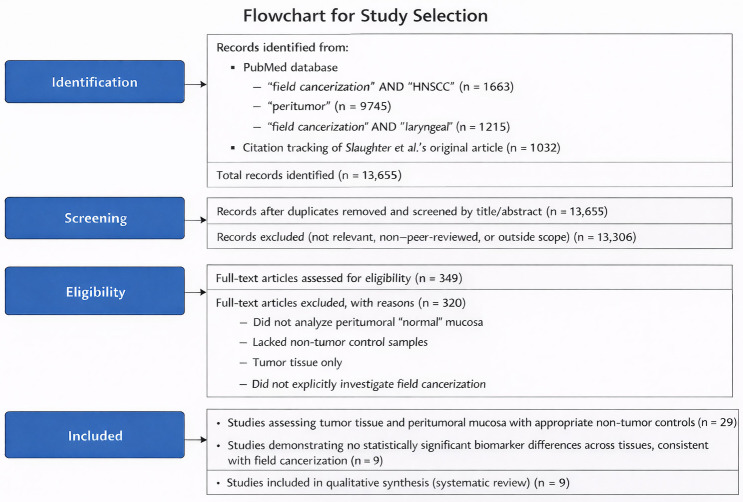
Flowchart illustrating the literature search and study selection process.

The inclusion criteria were restricted to studies that explicitly investigated field cancerization by analyzing not only tumor tissue, but also peritumoral “normal” mucosa, together with an additional control group of non-tumor samples Figure 1. This study design provides the necessary framework for evaluating the peritumoral field and thereby enables assessment of field cancerization at the molecular level. After removal of duplicates, records were screened by title and abstract, followed by full-text assessment based on predefined inclusion criteria focusing on studies comparing tumor tissue, peritumoral mucosa, and appropriate non-tumor controls. The study selection process and reasons for exclusion are summarized in a flowchart Figure 2. Despite the breadth of the search strategy, only nine publications met these stringent criteria, highlighting a notable gap in the literature regarding the molecular mechanisms of field cancerization in HNSCC. Through an extensive literature review, we identified nine studies that investigated potential biomarkers and met our predefined inclusion criteria Table 1.

Records were identified through a literature search of the PubMed database using predefined keywords related to field cancerization, peritumoral tissue, and head and neck squamous cell carcinoma, supplemented by citation tracking of the original study by Slaughter et al. Following title and abstract screening, full-text articles were assessed for eligibility based on predefined inclusion criteria, with reasons for exclusion documented. A total of nine studies met the criteria and were included in the qualitative synthesis.

Molecular genetic approaches have recently challenged the notion that independent transforming events are common in the epithelium of HNSCC patients. Indeed, when a primary HNSCC is compared with a second tumor elsewhere in the respiratory tract, paired tumors often harbor identical patterns of genetic alterations [9,10]. Understanding the molecular mechanisms underlying field cancerization is crucial for elucidating the initiation and progression of pre-neoplastic lesions. The transition from normal epithelium to premalignant tissue and finally to carcinoma is in part caused by a summation of genetic and epigenetic modifications. Epigenetics refers to changes in DNA expression that are not attributable to alterations in DNA sequence. The main events responsible are DNA methylation, histone modification, and post-transcriptional gene downregulation by microRNAs (miRNAs). These alterations can persist for the lifetime of a cell and can be inherited by subsequent generations.

The results of epigenetics vary from increased gene expression to complete silencing, depending on the interference of these alterations with activators and suppressors of specific promoters. Therefore, epigenetic changes can induce the overexpression of oncogenes as much as the silencing of tumor suppressor genes [11]. DNA methylation is the most common epigenetic event [12]. It is catalyzed by a series of enzymes known as DNA methyltransferases (DNMTs). These enzymes catalyze the covalent addition of a methyl group to the carbon-5 position of cytosine bases located 5′ to a guanosine base in a CpG dinucleotide [13]. Studies have found that only approximately 2% of the human genome encodes proteins, while the remaining 98% encodes different classes of non-coding RNA (ncRNAs) that were considered “junk” until recently. Among these are genes responsible for the expression of microRNAs (miRNAs) and long non-coding RNA (lncRNAs). Many of these ncRNAs have regulatory functions at the transcriptional, post-transcriptional, and epigenetic levels, and often display altered regulation in malignant neoplasia.

Evidence for peritumoral mucosal dysregulation and field cancerization includes differences in the proliferative index, morphometric variation in nuclear and cytoplasmic areas, chromosomal aberrations, microsatellite alterations, P53 expression, and altered CK expression in epithelial cells, which emphasizes the possibility that these molecular alterations can provide mechanisms for limitless replicative capacity and genomic instability that support tumor initiation and progression [5,14,15].

**Figure 3:**
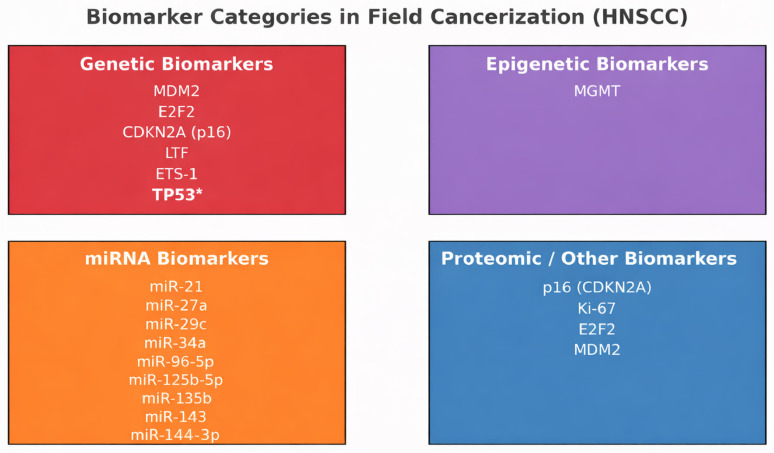
Categories of biomarkers implicated in field cancerization in HNSCC. Genetic, epigenetic, miRNA, and proteomic biomarkers associated with dysregulation across affected mucosal fields are summarized. * The TP53 gene is included as a paradigmatic molecular hallmark of field cancerization and clonal evolution rather than as a candidate biomarker identified through tripartite tumor–peritumor–control comparisons.

From our extensive literature review, the following genes, mRNAs, and proteins have emerged as potential biomarkers for field cancerization in head and neck cancer, and they represent the only candidates that met the predefined study design criteria (Table 2, Figure 3).

**Table 1:** Studies reporting potential biomarkers of peritumoral mucosal dysregulation that use the tripartite tumor–peritumor–control comparison.

Biomarker	Method	Stage	Localization	HPV Status	Ref.
**CDKN2A, MDM2, E2F2 AND LTF**	RT-qPCR	T1-4	oral cavity	N/A	[16]
**E2F2, MDM2 AND P16**	ELISA	T1-4	tongue, gums, floor of mouth	Mixed	[17]
**ETS-1**	RT-qPCR	T3-4	larynx	HPV-negative	[18]
**MGMT**	methylation-specific PCR (MSP)	T1-4	oral cavity	Mixed	[19]
**HSA-MIR-221, HSA-MIR-21, HSA-MIR-135B, AND HSA-MIR-29C**	RT-qPCR	N/A	oral cavity	N/A	[20]
**MIR-21, MIR-27A, MIR-146A, MIR-34A, MIR-143**	RT-qPCR	II-IV	oro/hypopharynx	N/A	[21]
**MIR-96-5P, MIR-21-3P, MIR-21-5P, MIR-429, KI-67**	RT-qPCR	N/A	oral cavity, hypopharynx, larynx	HPV-negative	[22]
**MIR-125B-5P, MIR-214-5P, E2F2 GENE AND PROTEIN**	RT-qPCR	T1-4	oral cavity	Mixed	[23]
**MIR-144-3P, MIR-145-5P**	RT-qPCR	T1-4	larynx	N/A	[24]

**Table 2:** List of the genes, microRNAs, and proteins reported as potential biomarkers. * The TP53 gene is included as a paradigmatic molecular hallmark of field cancerization and clonal evolution rather than as a candidate biomarker identified through tripartite tumor–peritumor–control comparisons.

Biomarker	Function	Tumorigenesis Role	Ref.
Genes			
TP53 *	DNA repair	Tumor suppressor, mutations lead to loss of cell cycle arrest	[25,26]
MDM2	p53 degradation	Oncogene, inhibits tumor suppressor p53	[16]
E2F2	Cell cycle progression	Promotes proliferation	[16,23]
CDKN2A	Cell cycle inhibition	Tumor suppressor, inactivated	[16]
LTF	Immune modulation, iron homeostasis	Tumor suppressor, downregulated in cancer	[16]
ETS-1	Transcription factor, angiogenesis	Oncogene, promotes metastasis	[18]
MGMT	DNA repair	Methylation can lead to accumulation of mutations	[19]
microRNA			
miR-21	OncomiR	Anti-apoptotic and PTEN suppression	[20,21,22]
miR-27a	OncomiR	Promotes drug resistance	[21]
miR-29c	Suppressor	Epigenetic regulator	[20]
miR-34a	Suppressor	Apoptosis, cell cycle arrest	[21]
miR-96-5p	OncomiR	Promotes proliferation	[22]
miR-125b-5p	Dual	Suppressor or oncogene (context)	[23]
miR-135b	OncomiR	Wnt and Hippo pathway suppression	[20]
miR-143	Suppressor	Anti-proliferative	[21]
miR-144-3p	Suppressor	Inhibits growth and metastasis	[24]
miR-145-5p	Suppressor	Targets stemness, downregulated	[24]
miR-146a	Dual	Immune modulator	[21]
miR-214-5p	Dual	Often suppressor	[23]
miR-221	OncomiR	Cell cycle progression	[20]
miR-429	Suppressor	Inhibits EMT	[22]
Proteins			
p16	CDK inhibitor, halts G1/S progression	Tumor suppressor; often lost in cancer	[17]
Ki-67	Proliferation marker (no direct functional role)	Diagnostic/prognostic marker for tumor aggressiveness	[22]
E2F2	Transcription factor for S-phase entry	Oncogenic; when overactive it promotes proliferation	[17,23]
MDM2	E3 ligase, inhibits/degrades p53	Oncoprotein; inhibits apoptosis and DNA damage response	[17]

### 3.1. Genomic Biomarkers

#### 3.1.1. p53

Mutations in TP53 (p53) serve as one of the most compelling molecular indicators of field cancerization in head and neck squamous cell carcinoma (HNSCC). The concept of field cancerization was first introduced by Slaughter et al. (1953) [1], who demonstrated that histologically normal epithelium adjacent to tumors often harbors early premalignant changes, suggesting a “condemned mucosa” predisposed to malignant transformation. In line with this pioneering observation, later molecular studies identified p53 mutations within histologically normal mucosa adjacent to HNSCC, establishing them as clonal markers of altered fields (Mohan et al., 2015) [25]. Meta-analyses have shown that TP53 mutations correlate with significantly poorer survival outcomes in HNSCC, underscoring their prognostic value (Basyuni et al., 2022) [26]. Collectively, these findings position TP53 as a paradigmatic molecular hallmark of field cancerization and clonal evolution. While TP53 is not included among candidate biomarkers derived from the comparative tripartite analyses, it is shown here to reflect its central role in clonal expansion and molecular field formation in HNSCC.

#### 3.1.2. MDM2 (Murine Double Minute 2)

In their paper, Gołąbek et al. compared the mRNA expression levels of MDM2 in tumor and margin samples from patients with cancers of the oral cavity [16]. The Mann–Whitney U test and Kruskal–Wallis test for statistical analysis and RT-qPCR showed no statistically significant differences in the expression levels. These results were replicated when, in another paper by Świętek et al., they tested the concentration of the MDM2 protein in the tumor and margin with the ELISA method [17]. They also found no statistically significant difference between the two tissues, displaying a qualitative abnormal similarity between the tissue samples and marking MDM2’s potential role as a biomarker for field cancerization.

MDM2 is an important negative regulator of the tumor suppressor protein p53. It encodes an E3 ubiquitin ligase that binds p53 and promotes its ubiquitination and subsequent proteasomal degradation. Under normal physiological conditions, MDM2 plays a critical role in maintaining p53 at low levels, thereby preventing unnecessary cell cycle arrest or apoptosis [27]. Elevated MDM2 levels have been found in many cancers, such as lung cancer, breast cancer, liver cancer, esophagogastric cancer, colorectal cancer, sarcomas, osteosarcomas, gliomas, melanomas, and hematopoietic malignancies [28,29]. In the context of cancer, particularly head and neck squamous cell carcinoma (HNSCC), overexpression or amplification of MDM2 has also been observed and can disrupt this regulatory balance. Elevated MDM2 levels lead to excessive degradation of p53, effectively disabling one of the most crucial cellular mechanisms for detecting and repairing DNA damage. As a result, cells with genomic instability can survive and proliferate, contributing to tumor initiation and progression [30,31]. The presented evidence points to MDM2 as a potential genomic and proteomic biomarker for field cancerization in HNSCC.

#### 3.1.3. E2F2 (E2F Transcription Factor 2)

Similarly to the MDM2 gene and protein, Gołąbek et al. [16] and Świętek et al. [17] tested the expression of the E2F2 gene and the concentration of the E2F2 protein in tumoral and peritumoral mucosa in oral cancer patients and reported no statistically significant difference in expression levels as both displayed a significant degree of dysregulation when compared to distant control sample. Thus, they identified an expression pattern that could be used as a biomarker for field cancerization.

E2F2 is a member of the E2F (Early region 2 binding factor) family of transcription factors that plays a pivotal role in the regulation of the cell cycle, particularly during the transition from the G1 to S phase [32]. E2F2 exerts its function primarily by regulating the expression of genes that are essential for DNA replication and cell cycle progression. E2F2 has been shown to have complex and context-dependent roles in tumorigenesis. E2F2 overexpression can contribute to oncogenesis by promoting unchecked cellular proliferation, a hallmark of cancer. E2F2 has been shown to interact with a variety of proteins involved in cell cycle regulation and DNA repair, including retinoblastoma protein (Rb), p53, and Breast Cancer gene 1 (BRCA1) [33,34,35,36]. E2F2 also plays a dual role in cancer, functioning not only as an oncogene but also as a tumor suppressor under certain conditions. Additionally, Li et al. documented that E2F2 variants are predictive biomarkers for recurrence risk in patients with OPSCC (oropharyngeal squamous cell carcinoma) [37]. E2F2′s versatile role in tumorigenesis and its similarity in expression in tumor and peritumor tissues make it a useful biomarker, and its link to field cancerization needs to be researched in future studies.

#### 3.1.4. CDKN2A

In their study, Gołąbek et al. evaluated and compared the gene expression level of CDKN2A in samples from tumor and peritumoral mucosa in HNSCC patients [16]. Using RT-qPCR and implementing the Mann–Whitney U test and Kruskal–Wallis test, they reported no statistically significant difference in expression levels, with similar levels of dysregulation, thereby making it eligible for our list of potential biomarkers for field cancerization in HNSCC. This gene encodes the distinct tumor suppressor protein p16^INK4a, which plays a critical role in regulating cell cycle progression and maintaining genomic integrity [38].

p16^INK4a functions by inhibiting cyclin-dependent kinases 4 and 6 (CDK4/6), thereby preventing phosphorylation and subsequent inactivation of the Rb protein. This inhibition keeps the Rb protein in its active, hypophosphorylated form, allowing it to sequester E2F transcription factors and block cell cycle progression from the G1 to S phase. As such, p16^INK4a serves as a key checkpoint regulator that ensures proper cell cycle control. The absence of functional p16^INK4a removes the critical inhibition of CDK4/6, leading to uncontrolled cell proliferation [39,40,41]. Loss or inactivation of CDKN2A through point mutations, deletions, or epigenetic silencing is one of the most frequent events in a wide range of human cancers [42,43,44,45,46]. Given its dual regulatory function through both RB and p53 pathways, CDKN2A is considered a central node in cell cycle control and tumor suppression. Its frequent alterations in cancer underscore its importance as a potential biomarker.

#### 3.1.5. LTF

In their paper, Gołąbek et al. also studied the gene expression levels of LTF in tumor tissue and NAT from HNSCC patients and reported statistically similar abnormal expression patterns in both samples, making it a potential biomarker for field cancerization in HNSCC [16]. This gene encodes lactotransferrin (also known as lactoferrin), an iron-binding glycoprotein that plays a key role in the regulation of immune responses, iron homeostasis, and inflammation. Although the biological functions of the lactotransferrin protein have been extensively characterized, the transcriptional regulation and expression patterns of the LTF gene are increasingly recognized as important in the context of tumorigenesis [47,48,49].

LTF plays a multifaceted role in maintaining epithelial integrity and modulating the tumor microenvironment. Gene expression studies have demonstrated that LTF is frequently downregulated in various malignancies, most notably in breast and nasopharyngeal cancers [50,51,52,53]. Functionally, loss of LTF expression correlates with enhanced cellular proliferation, reduced apoptosis, and increased metastatic potential in tumor cells. Conversely, re-expression of LTF has been shown to inhibit tumor growth in experimental models, suggesting that it may act as a tumor suppressor gene. Its anti-tumor effects are thought to be mediated, at least in part, through the regulation of iron availability, modulation of immune signaling pathways, and suppression of pro-inflammatory and pro-oncogenic signaling cascades [54,55]. Given its broad functional repertoire and consistent downregulation in multiple types of cancer, LTF is a promising biomarker. Interestingly, Deng et al. reported that overexpression of another potential biomarker, miR-214, correlated with reduced LTF mRNA and protein levels in nasopharyngeal carcinoma [56]. Further investigation of the mechanisms governing LTF gene silencing and its downstream effects may yield valuable insights into cancer pathogenesis and treatment strategies.

#### 3.1.6. ETS-1

In their search for the interconnection between the peritumoral mucosa and the process of tumor neoangiogenesis, Popov et al. applied RT-qPCR to samples from tumors and peritumoral mucosa, and reported that the ETS-1 molecule was abnormally overexpressed to a similar extent in both the tumoral and peritumoral mucosa of patients with laryngeal cancer (45% vs. 42%), and no statistically significant difference was found [18]. Their results showed a promising molecular signature that can be associated with field cancerization.

ETS-1 encodes a member of the ETS (E26 transformation-specific) family of tran-scription factors, which are key regulators of diverse biological processes, including cell development, proliferation, angiogenesis, and the formation of new blood vessels, which is critical for supplying nutrients and oxygen to rapidly growing tumors [57]. In tumorigenesis, ETS-1 is frequently upregulated in a range of cancer types [58,59,60]. Because of its central role in regulating oncogenic pathways, ETS-1 is considered a marker of tumor aggressiveness and a potential biomarker for field cancerization.

#### 3.1.7. MGMT (O-6-Methylguanine-DNA Methyltransferase)

Kato et al. tested the promoter hypermethylation rates of MGMT in tumor, margin, and control tissues from patients with oral cavity cancers [19]. RT-qPCR and Fisher’s exact test for statistical analysis showed no statistically significant differences between tumor and margin samples, whereas no methylation was detected for MGMT in normal mucosa from healthy volunteers. Their report revealed a strong quantitative similarity between tumor and adjacent tissue in contrast to control samples and indicated MGMTs’ strong potential of MGMTs as a biomarker for field cancerization in oral squamous cell carcinoma (OSCC).

The MGM gene encodes a critical DNA repair enzyme that plays a vital role in maintaining genomic integrity by removing mutagenic and cytotoxic adducts from the O6 position of guanine in the DNA. Specifically, MGMT repairs the damage caused by alkylating agents, a class of chemotherapeutic drugs commonly used to treat various cancers [61]. By repairing these lesions, MGMT helps prevent mutations and maintains genome stability, which is crucial for normal cellular function. In the literature, MGMT is frequently inactivated in colon, lung, and liver tumors and is strongly linked to tumorigenesis [62,63,64].

### 3.2. Transcriptomic Biomarkers

MicroRNAs (miRNAs) are a recently discovered class of small non-coding RNAs that regulate gene expression [65]. The first reported oncomiR in the literature was described by Calin et al. in 2002 in B-cell chronic lymphocytic leukemia cells [66]. Since then, numerous microRNAs have become a major focus in cancer research because of their association with tumor proliferation, invasion, and other hallmarks of cancer. A number of research groups have identified unique and attractive candidate miRNAs as biomolecule-based markers.

#### 3.2.1. MiR-21

MiR-21 is one of the most extensively studied microRNAs (miRNAs) in cancer bi-ology and is widely recognized as a prototypical oncomiR [67]. It is an abundantly ex-pressed miRNA in mammalian cells, and its upregulation is associated with numerous types of cancer [68,69]. MiR-21 was found to be the only consistently upregulated miRNA in a study that profiled 540 clinical samples from cancer patients [70].

Using qRT-PCR, two separate papers authored by Lopes et al. [20] and Orosz et al. [21] showed similar abnormal patterns of miR-21 expression in tumor and peritumor samples from patients with HNSCC compared to control epithelium samples. Ganci et al. reported intermediate expression levels in peritumoral tissue, with a rising gradient from normal mucosa to the highest expression in the tumor, demonstrating an expression pattern emblematic of biomarkers associated with field cancerization [22].

Functionally, miR-21 exerts oncogenic activity by targeting and downregulating multiple tumor suppressor genes. The most well-characterized targets are phosphatase and tensin homolog and PDCD4 (programmed cell death 4) [71,72,73]. Through the repression of these genes, miR-21-5p contributes to several hallmarks of cancer, including inhibition of apoptosis, enhancement of cell survival, increased proliferation, and promotion of cell migration and invasion. Ubiquitous overexpression across cancer types and its functional importance in multiple oncogenic pathways is one of the most critical miRNAs to be studied as a potential biomarker in HNSCC.

#### 3.2.2. MiR-27a

Orosz et al. used RT-qPCR to study mRNA expression in samples from patients with pharyngeal cancer [21]. For miR-27a, they reported a gradual change in the degree of dysregulation in the tumor and surrounding tissue in the meso- and hypopharyngeal tissue and tumor-adjacent tissues, thereby highlighting the expression pattern characteristic of a biomarker for field cancerization.

MiR-27a exerts its effects through the post-transcriptional regulation of tumor suppressor genes. Two of its well-characterized targets are FOXO1 (Forkhead box O1) and DROSHA/DGCR8 [74,75,76,77]. It is widely implicated in cancer development and progression and functions predominantly as an oncomiR in various tumor types. It is frequently overexpressed in malignancies, such as hepatocellular, colorectal, esophageal, and gastric cancers, where it contributes to several oncogenic processes, including enhanced proliferation, metastasis, and therapeutic resistance [75,76,78].

#### 3.2.3. MiR-29c

In another study conducted by Lopes et al., the expression of miR-29c was inves-tigated in various tissue samples, including noncancerous, adjacent to the tumor, and cancerous tissues [20]. They found that miR-29c expression levels were notably altered in normal and adjacent-to-tumor tissues, suggesting its potential involvement in the early stages of carcinogenesis or tumor progression. Moreover, no significant differences were observed in miR-29c expression between cancerous and adjacent tissues, highlighting the quantitative similarity that can be used in the search for cancerized fields.

MiR-29 is involved in the regulation of cell growth, apoptosis, differentiation, and proliferation, and its dysregulation has been linked to cancer progression. Evidence suggests that mir-29 functions both as a tumor suppressor and, context dependently, as an oncogenic factor, influencing extracellular matrix remodeling, epithelial–mesenchymal transition, and resistance to apoptosis—key events in HNSCC development [79]. It has been linked to the development of various cancers, including lung [80,81] and ovarian cancers [82]. These findings have made miR-29 a biomolecule of interest in future studies as a biomarker of field cancerization.

#### 3.2.4. MiR-34

In their study, Orosz et al. showed an inverse pattern of expression for miR-34, being the lowest in the tumor tissue and higher in the tumor-surrounding tissues in patients with cancer of the oro/hypopharynx [21]. Another interesting paper is that by Metheetrairut et al., who showed a connection between TP53 mutations and mir-34 [83]. Using qRT-PCR, they compared the expression patterns of the mir-34 family members in adjacent tumor tissues from patients with HNSCC with and without TP53 mutations. They reported that miR-34 family miRNAs were expressed at higher levels in tumor samples than in adjacent tumor tissues in a subgroup with wild-type TP53. In contrast, the data in the subgroup of patients with TP53 mutations undoubtedly displayed that miR-34a, miR-34b, and miR-34c had similar levels of expression to their matching samples of peritumoral mucosa. Thus, Metheetrairut et al. presented a possible link between two potential biomarkers, showing that miR-34 behaved as a biomarker for field cancerization only in tumors with mutations in the TP53 gene.

MiR-34 is a critical tumor suppressor microRNA, known for its involvement in regulating key cellular processes such as apoptosis and cell cycle arrest, and has been found to be dysregulated in various cancers [84,85,86]. It is also the first miRNA that was demonstrated to be directly regulated by the tumor suppressor p53 [87]. An increasing number of studies have illustrated the negative regulation of miR-34 family members in tumor cell metastasis and invasion [88,89], indicating an important relationship between the miR-34 family and EMT.

In the context of field cancerization in head and neck squamous cell carcinoma, Ganci et al. provided compelling evidence of miRNA dysregulation in peritumoral tissues [22]. Their research demonstrated that the miRNA expression pattern in peritumoral tissues was intermediate between that in tumoral and distant control tissues, suggesting early molecular alterations preceding overt tumor formation.

#### 3.2.5. MiR-96-5p

Mir-96 is one of the miRNAs identified in this study owing to its oncogenic properties and its role in modulating key tumor suppressor pathways. MiRNA-96 is a member of the miR-183-96-182 cluster [90,91]. It plays an important role in the regulation of the biological behavior of cancer cells [92,93,94,95]. Interestingly, Ganci et al.’s findings indicate that the expression of miR-96-5p in peritumoral tissues is not as elevated as in tumoral tissues, but is higher than that in normal control tissues [22]. This intermediate expression pattern underscores the potential of miR-96-5p as an early biomarker for HNSCC and highlights its involvement in the molecular landscape of field carcinogenesis. The aberrant expression of miR-96-5p in histologically normal-appearing peritumoral tissues suggests that these regions harbor molecular alterations that predispose them to malignant transformation. Collectively, these insights into miR-96-5p’s role in HNSCC pathogenesis emphasize the importance of considering peritumoral tissues in cancer research and developing early detection strategies. The intermediate expression of miR-96-5p in peritumoral areas offers a window into the early molecular events of tumorigenesis, providing opportunities for targeted interventions aimed at preventing progression to full-blown malignancy.

#### 3.2.6. MiR-125-5p

Gołbek et al. studied the expression levels of this mRNA in oral squamous cell carcinoma tumor and margin samples. By applying RT-qPCR and the Mann–Whitney U test for statistical analysis, they showed that miR-125b-5p was downregulated in both, with no statistically significant difference in the levels of expression [23]. They also reported a difference in the expression levels between smokers and non-smokers, alluding to the idea of epigenetic changes that occur from environmental influences.

The miR-125 family is highly conserved and comprises a few homologs (e.g., miR-125a-3p, miR-125a-5p, miR-125b-1, and miR-125b-2) [96]. MiR-125b-5p is a versatile miRNA with a dual role in cancer biology, exhibiting both tumor-suppressive and oncogenic functions, depending on the context and tissue type. This miRNA regulates critical cellular processes, such as apoptosis, proliferation, and differentiation by targeting a range of genes, including PIK3CD [97], A20 [98], and BCL2 [99]. In certain types of cancer, such as nasopharyngeal cancer, miR-125b-5p acts as a tumor suppressor, inhibiting oncogenic pathways and promoting apoptosis. In this context, its downregulation or loss of function can contribute to tumorigenesis and disease progression. Conversely, in other cancers such as hepatocellular cancer, miR-125b-5p exhibits oncogenic properties, and its overexpression may drive tumor growth and metastasis by targeting key regulatory genes involved in apoptosis and cell survival [100]. This paradoxical behavior underscores the complexity of miR-125b-5p’s role in cancer, highlighting its context-dependent actions that can either suppress or promote tumorigenesis.

#### 3.2.7. Mir-135b

Lopes et al. discovered that has-miR-135b is expressed abnormally at higher levels in both cancer and surrounding tissue samples of OSCC than in non-cancerous tissues [20]. They reported that the degree of dysregulation in the peritumoral tissue was similar to that in the tumor and showed a significant difference between non-cancerous and adjacent tumor tissues in patients with OSCC. This expression pattern makes this short non-coding RNA molecule a potential biomarker for field carcinogenesis in HNSCC.

miR-135b is a prominent oncomiR that plays a key role in promoting cancer pro-gression by inhibiting the tumor suppressor pathways. Targeting the critical tumor suppressor miR-135b disrupts important cellular pathways that normally prevent uncontrolled cell growth and tumorigenesis [101]. It has been shown to play a role in tumorigenesis in various types of cancer, including gastric, pancreatic, colorectal, and others [102,103,104].

#### 3.2.8. MiR-143

In their paper, Orosz et al. showed a gradual increase in the expression level of miR-143 from the tumor towards tumor margins and the highest expression level in normal mucosa, 3 cm from the tumor [21]. These results demonstrated that miR-143 plays a crucial role in the early stages of tumorigenesis and displays the expression pattern of a gradient biomarker for field cancerization in HNSCC.

MiR-143 is a critical tumor suppressor miRNA involved in the regulation of cell proliferation and tumorigenesis [105]. It was indicated that miR-143 expression was considerably downregulated in different types of cancer, including cervical, lung, gastric, and bladder [106,107,108,109].

#### 3.2.9. MiR-144-3p

In their paper on the microRNA expression profile in laryngeal carcinoma, Popov et al. reported that the tumor suppressor genes miR-144-3p and miR-145-5p were significantly dysregulated in peritumor mucosa, with a pattern of expression consistent with paired tumor samples and a statistically significant quantitative difference from control samples, making them potential biomarkers for field cancerization in the larynx [24]. Functionally, miR-144-3p has been shown to impair tumor cell proliferation, invasion, and migration, while promoting apoptosis. Conversely, its loss enhances oncogenic signaling and facilitates cancer progression [110]. It is frequently downregulated in malignancies such as lung [111,112], gastric [113], and esophageal malignancies [114], and its reduced expression is commonly associated with enhanced tumor aggressiveness and poor clinical outcomes.

#### 3.2.10. MiR-145-5p

MiR-145-5p is a well-established tumor suppressor miRNA that plays a critical role in inhibiting cancer cell proliferation, migration, and maintenance of cancer stem cell–like properties [115]. It is commonly downregulated in multiple tumor types, including lung [116], breast [117], gastric [118], and prostate malignancies [119]. Given its consistent downregulation and influence on critical oncogenic pathways, miR-145-5p has the potential to become a biomarker in the search for abnormal molecular premalignancies.

#### 3.2.11. MiR-146a

In their study, Orosz et al. studied tissues from pharyngeal carcinomas and reported a gradual decrease in the relative expression rate of miR-146a scaling from tumor tissues to normal mucosa, describing the typical quantitative expression pattern of a biomarker for field cancerization [21].

miR-146a is a versatile miRNA that plays a crucial role in the regulation of immune responses and inflammation. By targeting key signaling molecules that are involved in the activation of different crucial pathways, such as the NF-κB pathway, miR-146a helps control inflammatory processes and modulates the immune response. This regulation is particularly important for maintaining immune homeostasis and preventing excessive inflammatory reactions, which can contribute to cancer development [120,121].

The role of miR-146a in cancer is context-dependent, exhibiting both tumor suppressor and oncogenic functions, depending on the cancer type. Exogenous miR-146a expression increases the proliferation of several cells [122,123,124]. This contrasts with the fact that miR-146a is suppressed in gastric and hepatic carcinomas [125,126], and the downregulation of miR-146a is associated with the pathogenesis of hematopoietic malignancies [127]. Taken together, miR-146a seems to play a wide variety of roles in the regulation of different phenotypes of cancer and can target a wide range of different genes in various cellular microenvironments, which has led to controversy regarding its role in carcinogenesis. This paradoxical behavior underscores the complexity of miR-146a’s role in cancer, where its effects are highly dependent on the specific tumor context and molecular environment.

#### 3.2.12. MiR-214-5p

Gołbek et al. reported that miR-214-5p was upregulated in both tumor and margin samples without a statistically significant difference when comparing tissues from pa-tients with OSCC [23]. MiR-214-5p plays a significant role in the regulation of key pro-cesses involved in cancer progression, including cell migration, invasion, and EMT. By targeting several critical genes, such as PTEN and EZH2, miR-214-5p influences the signaling pathways that govern cellular movement, tissue remodeling, and metastasis [128]. MiR-214 is deregulated in several human cancers and displays contrasting behavior, suggesting that the cell context is crucial for miR-214 function. It is upregulated in pancreatic [70], prostatic [70], and gastric cancers [70,129]. However, when downregulated, it is associated with hepatocellular [130], bladder [131], and colorectal cancers [132]. The context-dependent behavior of miR-214-5p underscores its potential usefulness as a biomarker for field cancerization.

#### 3.2.13. MiR-221

MiR-221 is a well-established oncomiR that plays a crucial role in promoting cell cycle progression and tumorigenesis. Lopes et al. tested tumor and tumor-free margin samples from patients with OSCC and compared them to distant control samples. They reported significant differences in the level of miR-221 expression in control tissues and a similar degree of dysregulation in tumors and margins [20]. Given the reported results, miR-221 should be considered a candidate biomarker for field cancerization in HNSCC.

By targeting key cyclin-dependent kinase inhibitors, specifically CDKN1B (p27) and CDKN1C (p57), miR-221 disrupts the regulatory checkpoints that control cell cycle progression. This inhibition of cell cycle regulators leads to enhanced proliferation, allowing cancer cells to bypass critical growth control mechanisms and proliferate uncontrollably [133]. As an oncomiR, miR-221 is frequently overexpressed in a variety of cancers and contributes to tumor progression and aggressive behavior [134,135,136].

#### 3.2.14. MiR-429

Intriguingly, Ganci et al. demonstrated that in contrast to other mRNAs that showed gradual changes in their expression levels, miR-429 showed an identical degree of dysregulation in tumor and peritumor tissues compared to controls in patients with HNSCC [22], thus making this molecule a promising candidate as a biomarker for field cancerization.

miR-429 is a member of the miRNA-200 family, which is implicated in EMT and has been reported to be dysregulated in small cell lung carcinoma, colorectal cancer, nasopharyngeal cancer, and bladder cancer, suggesting that it may play a role in tumorigenesis and progression in different types of tumors [137,138,139,140]. MiR-429 is relevant to tumorigenesis in a tumor type-specific pattern, which might specifically function as either a tumor suppressor or tumor promoter candidate for certain cancers, depending on the particular type of tumor cells/tissues [140,141,142]. Therefore, miR-429 helps to maintain epithelial integrity and restricts the ability of cancer cells to migrate and invade the surrounding tissues. This tumor-suppressive function makes miR-429 a key molecule for future research in field cancerization.

### 3.3. Proteomic Biomarkers

#### 3.3.1. p16^INK4a

p16^INK4a, encoded by the CDKN2A gene, is a critical tumor suppressor protein that plays a central role in regulating the G1 phase of the cell cycle, and as a member of the INK4 family of cyclin-dependent kinase inhibitors, p16 functions by specifically in-hibiting CDK4 and CDK6 [38]. In their paper, Świętek et al. analyzed the quantitative expression of this protein in tumor and peritumor tissue with the ELISA method and found similar levels of abnormal quantities, thus showing that p16 is a potential biomarker that can be used to detect cancerized fields; further research should be performed to determine the importance of these results [17].

Beyond its canonical role in cell cycle arrest described in the paragraph on the CDKN2A gene, emerging evidence suggests that p16 may also influence cellular aging and responses to genotoxic stress, further highlighting its multifaceted role in tumor suppression. Given its influential role in tumorigenesis and association with HPV infections, p16 remains a critical focus in cancer biology and clinical pathology [143,144].

#### 3.3.2. Ki-67

In their study, Ganci et al. performed immunohistochemical analysis of tumor, peritumor, and control tissues from patients with head and neck cancer to test the concentration levels of Ki-67 as a proliferation marker and found that there was a statistically significant difference between the tumor and peritumor compared to the controls [22].

Ki-67 is a nuclear protein, originally identified by Gerdes et al. in the early 1980s [145]. It is encoded by the MKI67 (Marker of Proliferation Ki-67) gene, which is a nuclear protein expressed only in proliferating vertebrate cells and is widely recognized as a robust marker of cellular proliferation [146]. Unlike proteins that directly regulate the cell cycle, such as p16, Ki-67 does not control cell division; rather, it serves as an indicator of cell proliferation. It is expressed during all active phases of the cell cycle, G1, S, G2, and M, but is notably absent in dormant cells in the G0 phase. Elevated MKI67 expression has been observed in a wide range of malignancies, including lung, bladder, breast, cervical, and urothelial carcinomas, upper urinary tract cancers, lymphoma, and cervical cancer [147,148,149,150,151,152]. This unique expression pattern underpins its utility as a proliferation marker that could be used as an indicator of cancerized fields [153,154,155].

#### 3.3.3. E2F2, MDM and p16

By using the ELISA method to test the quantitative expression patterns of specific cancer-associated proteins in tumor and peritumor tissue, Świętek et al. found no statistically significant difference in the concentration of E2F2 protein across three separate articles [16,17,23], making it the most tested potential biomarker on our list. They also showed similar results for MDM2 and p16 [17]. These are some of the key proteins associated with the cell cycle [28,156,157]. However, the exact roles of E2F2, MDM2, and p16 in prognosis and biological function have not been well established in HNSCC, leaving a need for further research on this subject.

## 4. Discussion

The search for reliable biomarkers of peritumoral dysregulation represents a pivotal step toward establishing molecular margins in HNSCC. Conventional histopathological evaluation, although indispensable, remains limited in its ability to detect subclinical molecular alterations within histologically normal-appearing mucosa adjacent to the tumor. Recent studies have demonstrated that peritumoral epithelial tissue in head and neck squamous cell carcinoma (HNSCC) often exhibits genetic and epigenetic aberrations—such as promoter hypermethylation, miRNA dysregulation, and TP53 mutations—similar to those identified in the primary tumor [16,17,20,21,22,24].

These observations reinforce the concept of field cancerization, originally proposed by Slaughter and colleagues [1], which postulates that prolonged exposure to carcinogens induces widespread molecular alterations across the mucosal field, predisposing it to multifocal malignant transformation. This framework has since evolved into a molecular model of progressive dysregulation, encompassing both tumor and peritumor compartments [4,5,8].

Identifying shared biomarkers of dysregulation between tumor and peritumoral mucosa has profound clinical implications. Molecular alterations such as the abnormal expression of MDM2, E2F2, CDKN2A/p16, ETS-1, and microRNAs including miR-21, miR-96-5p, and miR-145-5p have been consistently detected in both tumor and adjacent non-tumorous tissues in patients with oral and laryngeal squamous cell carcinoma [16,18,19,20,23,24]. These shared molecular profiles delineate areas of biologically altered yet histologically unremarkable tissue that may extend beyond resection margins defined by routine microscopy.

The concept of a molecular margin—defined by the presence or absence of cancer-associated molecular alterations rather than by morphology alone—offers a more precise framework for assessing residual disease risk [158,159]. Incorporating biomarker-based molecular profiling into intraoperative or postoperative evaluation could improve surgical precision, guide adjuvant treatment, and minimize local recurrence. Thus, the study of peritumoral dysregulation not only deepens our understanding of field cancerization but also provides a translational bridge toward personalized oncologic surgery, where molecular diagnostics redefine the meaning of a “clear” surgical margin.

Beyond their mechanistic relevance, biomarkers of peritumoral dysregulation may hold clinical value as tools for risk stratification and disease monitoring in head and neck squamous cell carcinoma. The presence of shared molecular alterations in tumor and adjacent mucosa may help identify patients at increased risk of local recurrence or second primary tumors, even when surgical margins are histologically negative. In this context, biomarker-based profiling of peritumoral tissue could complement conventional staging and surveillance strategies, supporting more individualized postoperative follow-up and therapeutic decision-making. Although clinical implementation remains premature, these findings highlight the potential role of peritumoral biomarkers as adjunctive diagnostic and prognostic indicators.

Recent advances in multi-omics and spatially resolved technologies, including single-cell sequencing, spatial transcriptomics, and genome-wide DNA methylation profiling, offer promising tools for refining the concept of molecular margins in head and neck squamous cell carcinoma. These approaches enable high-resolution characterization of molecular gradients across tumor–peritumoral interfaces and can reveal field effects beyond histologically defined tumor borders. However, despite their relevance, the application of such methodologies to peritumoral dysregulation and field cancerization in HNSCC remains limited, with most studies relying on bulk tissue analyses and targeted biomarkers. As these technologies become more accessible, their integration into future research will be essential for improving the precision and clinical utility of molecular margin assessment.

An important limitation of the studies summarized in this review is the marked heterogeneity of patient cohorts, which may influence the interpretation of shared molecular dysregulation. As shown in Table 1, the included studies encompass multiple anatomical subsites, including the oral cavity, oropharynx, hypopharynx, and larynx, as well as a broad range of disease stages (T1–T4). In addition, HPV status was inconsistently reported, with only a minority of studies explicitly including HPV-positive cases, while most lacked HPV stratification altogether. This is particularly relevant given the distinct molecular characteristics of HPV-associated versus HPV-negative HNSCC, especially with respect to p53 pathway alterations. Environmental exposures such as tobacco and alcohol use were also variably documented. Collectively, these sources of heterogeneity likely contribute to variability in reported biomarker profiles and underscore the need for standardized reporting and stratified analyses in future studies.

In our literature review, we faced challenges when trying to collect a portfolio of papers with potential biomarkers. The first challenge was that there was a scarce number of papers written on this topic in terms of HNSCC. While performing the literature search with the keywords “peritumor”, it became apparent that research into field cancerization for HNSCC is far behind research for breast cancer, hepatocellular cancer, esophageal cancer, pancreatic cancer, gliomas, and many more. Another challenge was that most of the papers reported a very small cohort of patients, which did not allow for valid statistical data to be extracted. An example is the very interesting paper by Tan et al. [160] that tested transcriptomic convergence as a driver for field cancerization in patients with synchronous SCC of the aerodigestive tract but only used a cohort of three patients.

The biggest obstacle in our literature search was differentiating between the definitions that the authors applied to the term tumor-adjacent tissue and the lack of papers comparing them to normal control samples. There was a large difference in the harvesting distance from the tumor, which ranged in scale. In terms of controls, many studies did not use distant normal tissue for comparison or used tumor-adjacent tissue as controls. As De Assumpção et al. very accurately stated [158], if we search for cancer biomarkers by comparing cancer samples with adjacent-to-cancer samples, it will most likely provide markers of progression from cancer fields to cancer, instead of resulting in the discovery of the earliest markers of cancer, because normal tissues are not compared. Moreover, referring to adjacent-to-tumor samples as normal samples can lead to misinterpretations, including missing the identification of potential biomarkers expressed in both tumor and adjacent tumor tissues in patterns different from those in true normal tissues. What we learned from these differences is that we need a standardized, better-structured, and guideline-following study models for future research in this field.

Biomarkers associated with field cancerization hold substantial translational potential in head and neck squamous cell carcinoma (HNSCC), particularly for guiding surgical, surveillance, and prognostic decision-making. Molecular analysis of surgical margins—despite being histologically clear—has identified alterations such as promoter methylation and TP53 mutations indicative of residual field changes, offering opportunities to predict local recurrence and tailor adjuvant therapies [159]. Furthermore, surveillance strategies that integrate these molecular markers alongside conventional imaging are emerging as promising tools for earlier detection of tumor recurrence and second primary malignancies [161,162]. Though still largely exploratory, these approaches underscore the potential utility of field cancerization biomarkers in enhancing margin assessment, refining postoperative follow-up, and improving patient outcomes.

## 5. Conclusions

Peritumor mucosal dysregulation, as one of the consequences of field cancerization, represents a critical aspect of head and neck cancer biology that influences recurrence, treatment outcomes, and patient prognosis. Mounting evidence indicates that histologically normal peritumoral mucosa often harbors molecular alterations similar to those found in the tumor itself, suggesting that cancer progression occurs across a molecularly altered field rather than as an isolated lesion. Recognizing and characterizing these changes are essential steps toward the establishment of molecular margins—zones defined by the presence or absence of cancer-associated genetic, epigenetic, or transcriptomic signatures rather than by morphology alone.

The development of molecular margin assessment could significantly improve surgical precision, enabling the identification of residual dysregulated tissue that escapes conventional histopathological evaluation. Integrating validated biomarkers—such as deregulated miRNAs, methylation patterns, and altered protein expression profiles—into perioperative diagnostics may enhance prognostic accuracy and guide decisions regarding adjuvant therapy. Advances in high-throughput sequencing, spatial transcriptomics, and real-time molecular diagnostics hold particular promise for translating this approach into clinical practice.

## Data Availability

No new data were created or analyzed in this study.

## Abbreviations

The following abbreviations are used in this manuscript:

HNSCC	Head and Neck Squamous Cell Carcinoma DNA
mRNA	Messenger RNA
miRNA	MicroRNA
miR	MicroRNA (often used as a prefix for specific miRNAs)
DNA	Deoxyribonucleic acid
DNMTs	DNA methyltransferases
ncRNA	non-coding RNA
lncRNA	long non-coding RNA
NAT	Normal tissue adjacent to the tumor
RT-qPCR	Quantitative reverse transcription polymerase chain reaction
P53	Tumor Protein p53 (a tumor suppressor)
MDM2	Murine double minute 2
E2F2	E2F Transcription Factor 2
E2F	Early region 2 binding factor
TAT	Tumor-adjacent tissue
Rb	Retinoblastoma protein
BRCA1	Breast Cancer gene 1
CDKN2A	cyclin-dependent kinase inhibitor 2A
NAT	normal adjacent tissue
p16	A specific protein encoded by CDKN2A, often referred to as p16INK4a
INK4a	Inhibitor of cyclin-dependent kinase 4
CDK4/6	cyclin-dependent kinases 4 and 6
LTF	lactotransferrin/lactoferrin
TNM	Tumor, Node, Metastasis (staging system)
NPC	nasopharyngeal cancer
ETS	E26 transformation-specific
The MGMT	O-6-Methylguanine-DNA Methyltransferase
PTEN	phosphatase and tensin homolog
PDCD4	programmed cell death 4
FOXO1	Forkhead box O1
EMT	Epithelial–Mesenchymal Transition
TP53	Tumor Protein P53
PIK3CD	phosphatidylinositol-4,5-bisphosphate 3-kinase catalytic subunit delta
BCL2	B-cell leukemia/lymphoma 2
OSCC	Oral squamous cell carcinoma
OPSCC	Oropharyngeal squamous cell carcinoma
NF-κB	nuclear factor-kappa B
EZH2	Enhancer of zeste homolog 2
CDKN1B	Cyclin-dependent kinase inhibitor 1B
HPV	Human papillomavirus
Ki-67	Kiel 67
MKI67	Marker of Proliferation Ki-67
